# Comprehensive analysis of the cancer driver genes constructs a seven-gene signature for prediction of survival and tumor immunity in hepatocellular carcinoma

**DOI:** 10.3389/fgene.2022.937948

**Published:** 2022-08-09

**Authors:** Jun Zou, Wan Qin

**Affiliations:** ^1^ Department of Pediatrics, Tongji Hospital, Tongji Medical College, Huazhong University of Science and Technology, Wuhan, China; ^2^ Department of Oncology, Tongji Hospital, Tongji Medical College, Huazhong University of Science and Technology, Wuhan, China

**Keywords:** cancer driver gene, tumor microenvironment, prognosis, nomogram, hepatocellular carcinoma

## Abstract

Hepatocellular carcinoma (HCC) is a highly malignant and heterogeneous tumor with poor prognosis. Cancer driver genes (CDGs) play an important role in the carcinogenesis and progression of HCC. In this study, we comprehensively investigated the expression, mutation, and prognostic significance of 568 CDGs in HCC. A prognostic risk model was constructed based on seven CDGs (CDKN2C, HRAS, IRAK1, LOX, MYCN, NRAS, and PABPC1) and verified to be an independent prognostic factor in both TCGA and ICGC cohorts. The low-score group, which showed better prognosis, had a high proportion of CD8^+^ T cells and elevated expression of interferon-related signaling pathways. Additionally, we constructed a nomogram to extend the clinical applicability of the prognostic model, which exhibits excellent predictive accuracy for survival. Our study showed the important role of CDGs in HCC and provides a novel prognostic indicator for HCC.

## Introduction

Global cancer statistics show that primary liver cancer is the sixth most commonly diagnosed cancer and the third leading cause of cancer death worldwide in 2020 (Sung et al., 2021). The most common type of liver cancer is hepatocellular carcinoma (HCC). Owing to the specific phenotypes, most HCC patients are diagnosed at an advanced stage with extremely poor prognosis (El-Serag, 2011). Although significant progress has been made in the diagnosis and treatment of HCC, the survival rate for patients over 5 years has not improved (Bruix et al., 2014). Recent studies about the molecular biological characteristics and the tumor microenvironments have revolutionized the management of HCC patients, leading to a transition from traditional chemotherapy to novel target therapy and immunotherapy (Shen et al., 2010; El-Khoueiry et al., 2017; Kudo et al., 2018; Zhu et al., 2018). However, only a small fraction of HCC patients can benefit from these novel therapeutic options, and some patients inevitably suffer from drug resistance (Kimura et al., 2018). Thus, further efforts are still needed to excavate the underlying molecular biological mechanisms as well as to seek effective predictive measures for personalized therapy.

As is known, cancer is a genetic disease characterized by changes in the genome, genes, chromatin, and cellular levels (Brown et al., 2019). Mutations in driver genes support the acquisition of cancer hallmarks (Hanahan and Weinberg, 2000; Hanahan and Weinberg, 2011). Recently, the journal of *Nature Reviews Cancer* reported a compendium of 568 cancer driver genes (CDGs), which was identified from more than 28,000 tumors of 66 cancer types (Martinez-Jimenez et al., 2020). Mutations and aberrant expression of these genes may affect cell growth, proliferation, tumor occurrence, and progression (Leroi et al., 2003). Tumorigenesis is often associated with alterations in the tumor microenvironment. Alterations of CDGs may influence the tumor microenvironment and affect the response to immunotherapy. Studying CDGs offers a chance to develop accurate biomarkers for tumor prognosis and make decision on the therapeutic strategy.

In the current study, we systematically profiled the expression characteristics and mutation landscape of the 568 CDGs in HCC. We constructed a prognosis score based on seven CDGs, which could predict the survival of HCC patients and was validated to be an independent prognostic factor in different HCC cohorts. Our study underlined the importance of CDGs in HCC and provided a strategy for patient stratification for precise medication.

## Materials and methods

### Data collection

A total of 424 pieces of RNA-seq data of TCGA-LIHC were downloaded from the Cancer Genome Atlas (TCGA) database as a training set, which consists of 374 HCC samples and 50 controls. A total of 230 HCC patient cases with gene expression and complete clinical information from the International Cancer Genome Consortium (ICGC) dataset were used as a validation set. The gene mutation and clinical data of TCGA-LIHC were also downloaded from the TCGA database.

### Differentially expressed cancer driver gene identification

Differentially expressed CDGs between cancer and normal tissues were identified by Wilcoxon test with |log2 fold change (FC)| ≥1 and FDR (false discovery rate) < 0.05. The mutation pattern of differentially expressed genes was analyzed by the Maftools R package.

### Functional enrichment analyses

In order to explore the functions and signaling pathways of the differently expressed CDGs, the “clusterProfiler” R package was used to perform the Kyoto Encyclopedia of Genes and Genomes (KEGG) pathway and Gene Ontology (GO) enrichment analyses, with *p* < 0.05 and FDR < 0.05 being used as significance thresholds.

### Construction of gene signature

Univariate Cox regression analysis was performed to find CDGs significantly related to survival. Least absolute shrinkage and selection operator (LASSO) regression analysis was performed to further screen prognostic-related CDGs. Finally, the stepwise multivariate COX regression analysis was performed to find the optimal key prognostic-related CDGs and obtained standardized regression coefficients. The risk score of each patient was calculated by the following formula: Risk score = Expression of gene1 × Coefficient of gene1 + Expression of gene2 × Coefficient of gene2 + ... Expression of geneN × Coefficient of geneN. Patients were assigned into low-risk groups and high-risk groups by the median value of the risk score, and the Kaplan–Meier curve was plotted by the “Survival” R package. Moreover, the receiver operating characteristic (ROC) curves were generated by the “SurvivalROC” R package to determine the accuracy of the gene signature.

### Gene signature validation by the ICGC database

The 230 HCC patient cases from the ICGC database were utilized as an external validation dataset. The risk score of each sample was calculated by the formula presented above. The Kaplan–Meier curve and the ROC curve were plotted as described above.

### Functional biological, immune infiltration, and tumor stemness analysis

Gene set enrichment analysis (GSEA) was performed using the gene set “c2. cp.kegg.v7.5. symbols.gmt” and “c5. go.bp.v7.5. symbols.gmt” (Subramanian et al., 2005). The “GSVA” package was used to compare the immune-related pathways between the two subgroups (Hanzelmann et al., 2013). CIBERSORT was conducted to calculate the immune cell fraction among the samples in the TCGA-LIHC cohort (Newman et al., 2015). Tumor stemness was reported to be capable of evaluation by RNA stemness score (RNAss) based on mRNA expression (Malta et al., 2018). Correlation between risk score and RNAss was analyzed using Spearman rank-based testing. The R code used in this study is available in the supplementary file.

## Results

### Mutation landscape and functional analysis of the differentially expressed CDGs in HCC

**FIGURE 1:**
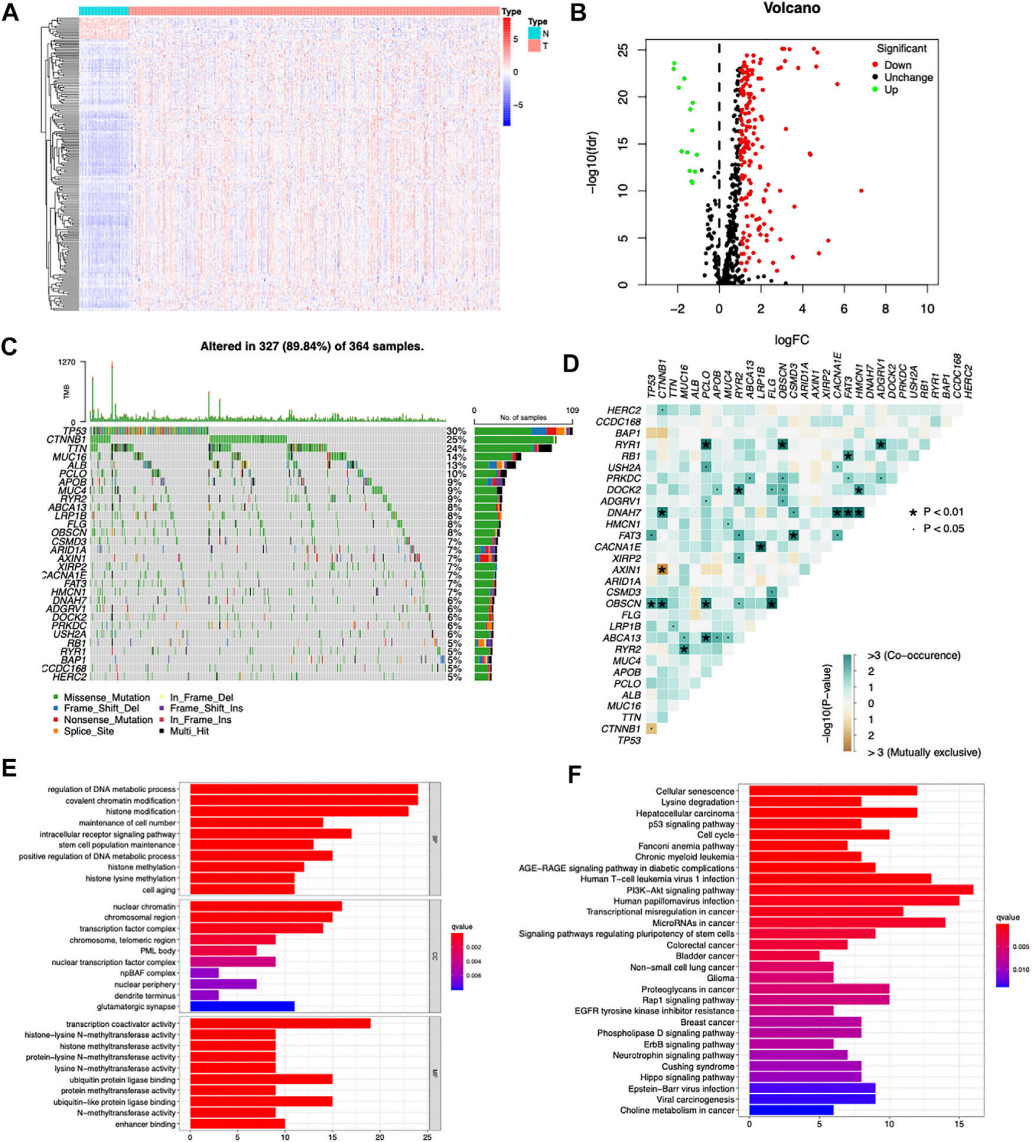
Searching differentially expressed CDGs in the TCGA cohort. **(A)** Heatmap of differentially expressed CDGs between HCC and normal tissues. **(B)** Volcano plot of differentially expressed CDGs between HCC and normal tissues. **(C)** Mutation spectrum of the top 30 most frequently mutated DE CDGs in the TCGA-LIHC cohort, with each column representing one patient and the percentage on the right side representing the corresponding gene mutation rate. **(D)** Interactions and crosstalk between mutated genes in the TCGA-LIHC cohort. **(E)** GO enrichment analysis of the upregulated DE CDGs. **(F)** KEGG enrichment analysis of the upregulated DE CDGs.

A total of 568 human CDGs obtained from the somatic mutations of more than 28,000 tumors of 66 cancer types were included in our study (Supplementary Table S1). By utilizing Wilcoxon test, 189 differentially expressed CDGs (DE CDGs) were identified, including 175 upregulated and 14 downregulated CDGs, according to the |log2 FC| ≥1 and FDR <0.05 (Figures 1A,B). Of note, most of the DE CDGs were highly expressed in the cancer tissues than in normal tissues.

Among the 189 differentially expressed CDGs, TP53 had the highest mutation rate (30%) followed by CTNNB1 (25%), TTN (24%), MUC16 (14%), ALB (13%), and PCLO (10%) (Figure 1C). Genetic interaction analysis identified that mutations of TP53 were positively correlated with mutations of FAT3 and OBSCN and negatively correlated with CTNNB1. Mutations of CTNNB1 were positively correlated with mutations of HERC2, DNAH7, and OBSCN and negatively correlated with AXIN1 (Figure 1D). These results indicated that CDGs had high mutation rates in HCC, and there were huge interactions and crosstalk between these mutations.

GO and KEGG pathway enrichment analyses were carried out to explore the functions and signaling pathways of the DE CDGs. GO enrichment analysis showed that the upregulated DE CDGs were mainly associated with “regulation of DNA metabolic process,” “covalent chromatin modification,” “histone modification,” and “histone methylation” of the biological process (BP) category. In the cellular component (CC) category, “nuclear chromatin,” “chromosomal region,” and “transcription factor complex” were significantly enriched. The molecular function (MF) term mainly included “transcription coactivator activity,” “ubiquitin protein ligase binding,” and “ubiquitin-like protein ligase binding” (Figure 1E). KEGG pathway enrichment analysis showed that upregulated DE CDGs were highly enriched in “PI3K-Akt signaling pathway,” “hepatocellular carcinoma,” “cell cycle,” and so on (Figure 1F). These results suggested that DE CDGs in the HCC play an important role in the key process in cancer development, such as epigenetic modification and various signaling pathways. These aberrantly expressed genes may lead to carcinogenesis and progression of HCC.

### Selection of prognosis-related CDGs

**FIGURE 2:**
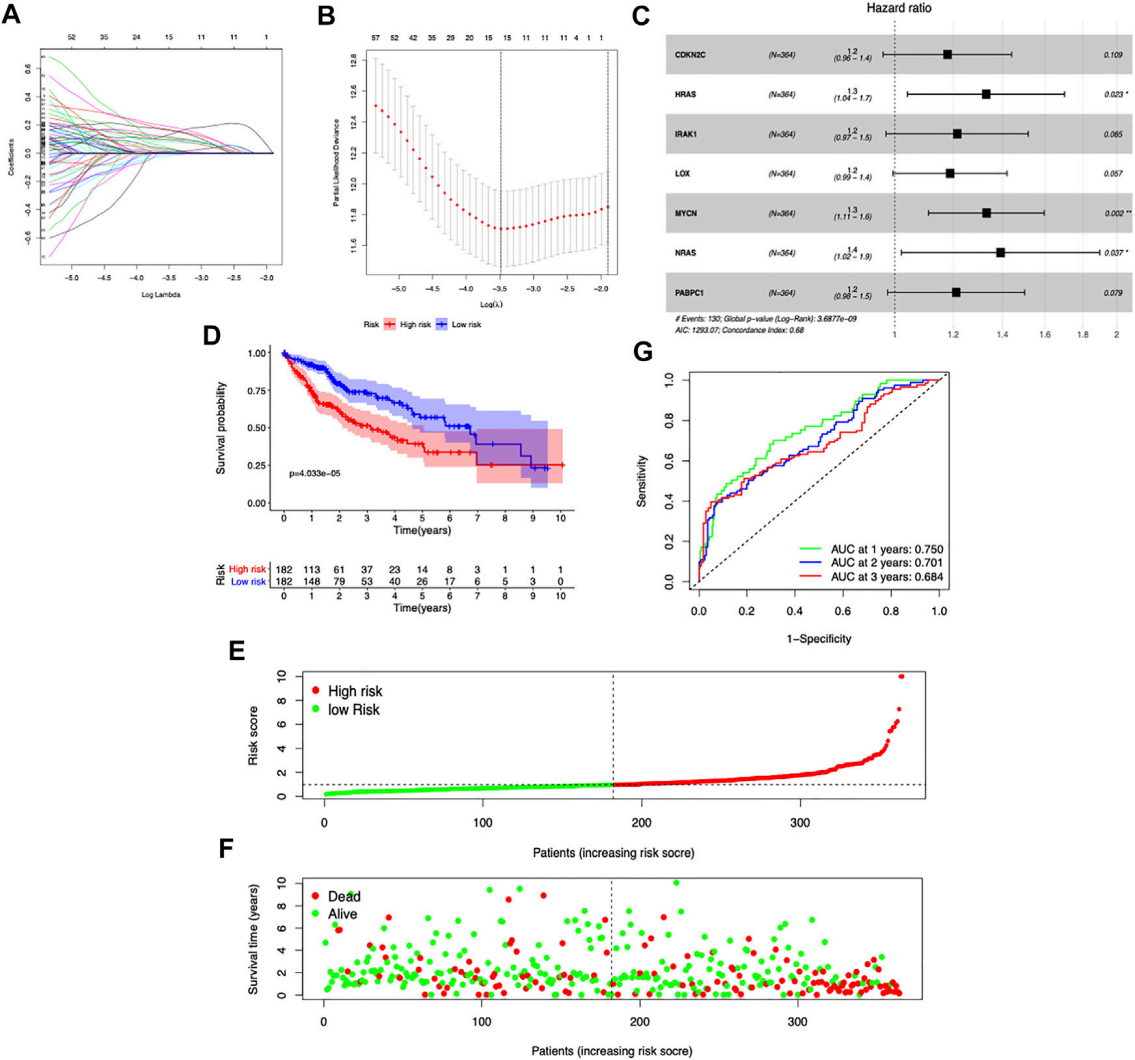
Construction of the prognostic risk model based on seven CDGs. **(A,B)** LASSO Cox regression analysis of the selection of CDGs. **(C)** Forest plot of the seven genes that construct the risk signature. **(D)** Survival curves stratified by the risk score in the TCGA cohort. **(E,F)** Distribution of risk score, survival time, and survival statuses in HCC patients. **(G)** Receiver operating characteristic (ROC) curves of risk model for predicting survival in the TGGA-LIHC cohort.

Among the 189 DE CDGs, 96 genes were found to be associated with the patients’ overall survival by univariate Cox regression analysis (Supplementary Table S2). Then, the LASSO regression analysis was conducted to further narrow the survival-related CDGs (Figures 2A,B). Finally, seven genes were identified by the stepwise multivariate regression analysis and subsequently used to construct a prognostic gene signature (Figure 2C). The seven genes identified were CDKN2C, HRAS, IRAK1, LOX, MYCN, NRAS, and PABPC1. The risk score = 0.1758* Expression of CDKN2C + 0.2975* Expression of HRAS +0.1934 * Expression of IRAK1 + 0.1943 * Expression of LOX + 0.3361 * Expression of MYCN + 0.3782 * Expression of NRAS + 0.2048 * Expression of PABPC1 (Supplementary Table S3).

To assess the prognostic capacity of the seven-gene signature, we calculated the risk score for each patient and classified the patients into low- and high-risk groups based on the medium risk score value. In the TCGA dataset, the overall survival rate of patients in the low-risk group was markedly higher than that of the high-risk group (*p* = 4.033e-5) (Figure 2D). As the risk score increased, the patients had a shorter survival time (Figures 2E,F). The area under the time-dependent ROC curves at 1-, 2-, and 3-year survival was 0.75, 0.701, and 0.684, respectively (Figure 2G). Taken together, our results suggested that the risk scores based on the seven CDGs had optimal prediction ability of the prognosis of HCC patients.

### Validation of the seven-gene signature in the ICGC dataset

**FIGURE 3:**
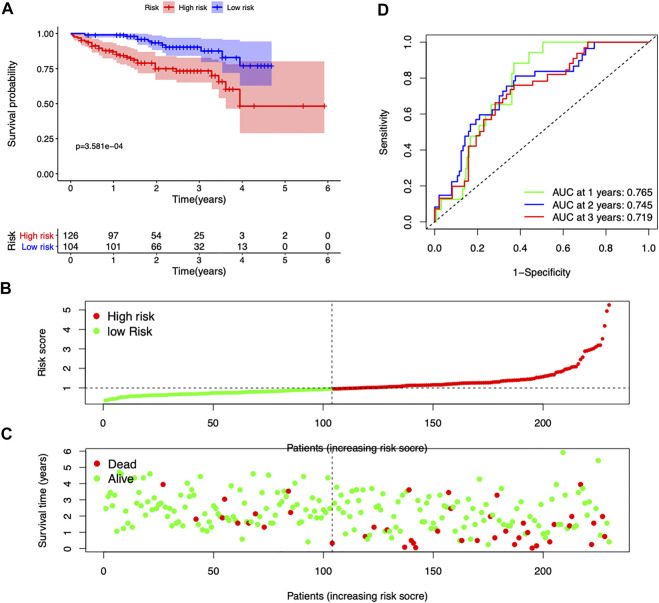
Validation of the seven-gene risk model in the ICGC dataset. **(A)** Survival curves stratified by the risk score in the ICGC cohort. **(B,C)** Distribution of risk score, survival time, and survival statuses in the ICGC cohort. **(D)** ROC curves of risk model for predicting survival in the ICGC cohort.

The ICGC database was used as a validation cohort to verify the accuracy of the seven-gene signature. Consistent with the TCGA dataset, high-risk score patients exhibited a significantly worse outcome (*p* = 3.581e-4) (Figure 3A). Patients with high-risk scores had more mortality and shorter survival time (Figures 3B,C). The time-dependent ROC curves suggested that the AUC at 1-, 2-, and 3-year survival was 0.765, 0.745, and 0.719, respectively (Figure 3D). These results suggested good accuracy and stability of our prognostic signature.

### Independent prognostic role of the prognostic signature

**FIGURE 4:**
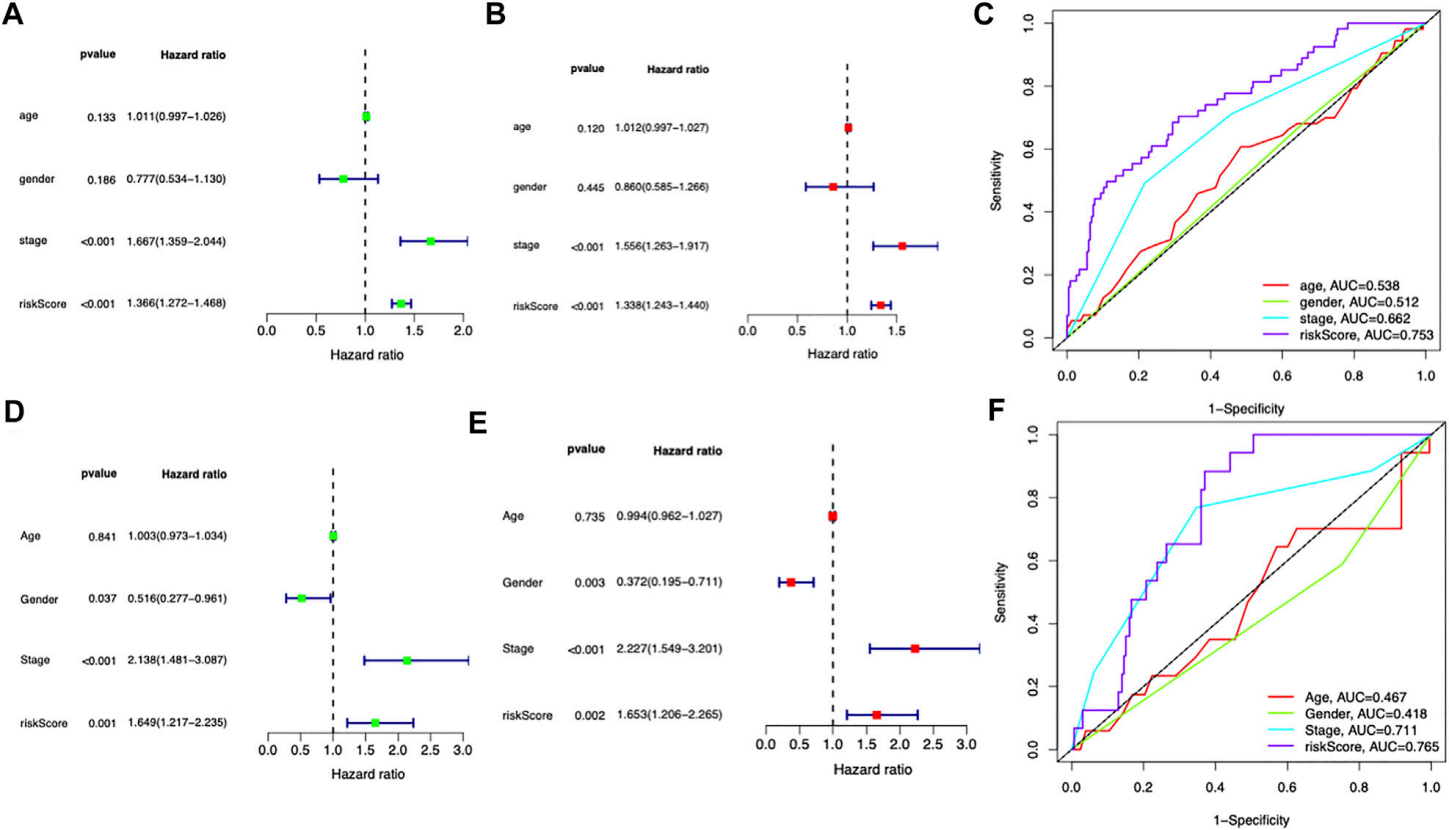
Independent prognostic role of the prognostic signature. **(A,B)** Univariate and multivariate Cox analyses for the prognostic model and other clinical features in the TCGA cohort. **(C)** ROC analysis of the risk score and other clinical features in the TCGA cohort. **(D,E)** Univariate and multivariate Cox analyses for the prognostic model and other clinical features in the ICGC cohort. **(F)** ROC analysis of the risk score and other clinical features in the ICGC cohort.

The clinical information including age, gender, and stage was included for further analysis both in the TCGA and ICGC cohorts. In the TCGA dataset, the risk score was independently associated with the survival of the patients, with a hazard ratio of 1.366 in the univariate analysis and 1.338 in the multivariate Cox regression (Figures 4A,B). In the ICGC cohort, the hazard ratio of the risk score was 1.649 and 95% confidence interval (CI) was 1.206–2.265 (*p* = 0.002) in the univariate Cox regression and 1.653 (1.206–2.656) in the multivariate regression (Figures 4D,E). The AUC of risk score was 0.753 in the TCGA cohort and 0.765 in the ICGC cohort (Figures 4C,F), which exceeded stage and other clinical features.

### Different cancer hallmarks and tumor microenvironments between two risk score groups

**FIGURE 5:**
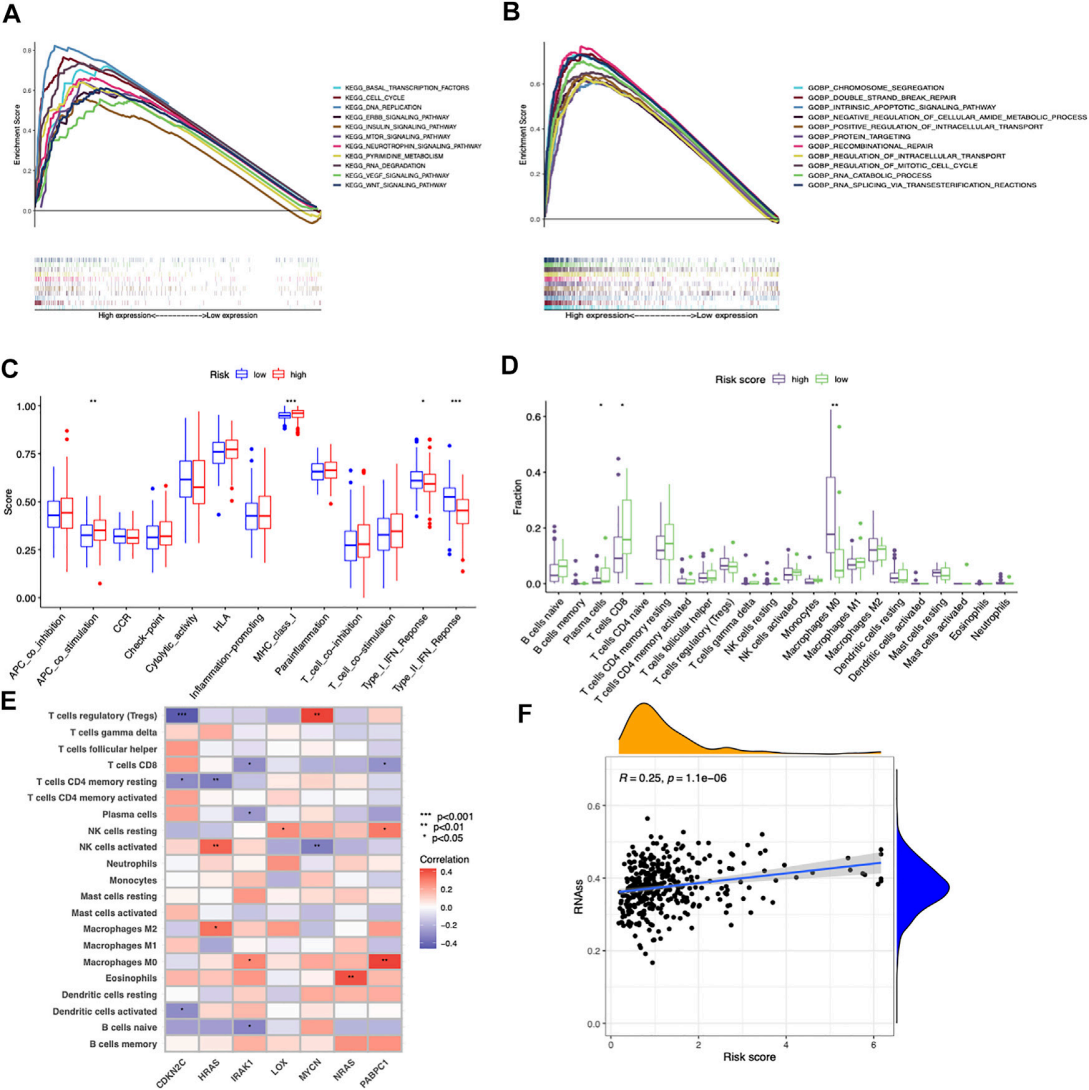
Different cancer hallmarks and tumor microenvironments between two risk score groups. **(A,B)** GSEA of significantly enriched pathways in the high-risk group based on KEGG and GO (biological process) gene sets. **(C)** Differences in the proportions of immune-related pathways between the low- and high-risk groups. **(D)** Differences in the immune cells infiltrated in the tumor microenvironment between the low- and high-risk groups. **(E)** Correlations between infiltrated immune cells and the seven prognostic-related cancer driver genes. **(F)** Correlation between risk score and cancer stemness score (RNAss) based on Spearman’s correlation tests.

GSEA was performed to explore the underlying biological processes related to the risk score signature. We found most cancer hallmarks, including the VEGF signaling pathway, cell cycle, DNA replication, ERBB signaling pathway, double strand break repair, positive regulation of intracellular transport, and regulation of mitotic cell cycle, were significantly enriched in patients in the high-risk score group (Figures 5A,B). However, immune-related pathways including Type_1_IFN_Response and Type_2_IFN_Response were significantly overexpressed in the low-risk score group (Figure 5C). Moreover, the infiltrating immune cell subtypes were significantly different between the high-risk group and the low-risk group, with more CD8+T cells and less M0 macrophages accumulating in low-risk score tumors (Figure 5D). Spearman’s correlation analysis showed that IRAK1 and PABPC1 expression was positively correlated with M0 macrophages and negatively correlated with CD8+T cells. CDKN2C showed negative correlation with Tregs, CD4 memory resting T cells, and activated dendritic cells. HRAS was positively correlated with M2 macrophages and activated NK cells (Figure 5E). The correlation between risk score with tumor stemness measured by RNAss was explored. The results showed that the risk score was significantly positively correlated with stemness score (*R* = 0.25, *p* = 1.1e-06, Figure 5F).

These results together implied that the longer OS of the low-risk score group might be attributed to an inflamed tumor microenvironment with more infiltrated CD8^+^ T cells and less M0 macrophages, while the poor prognosis of the high-risk score group might be associated with the tumorigenesis of cancer hallmarks.

### A predictive nomogram development and validation

**FIGURE 6:**
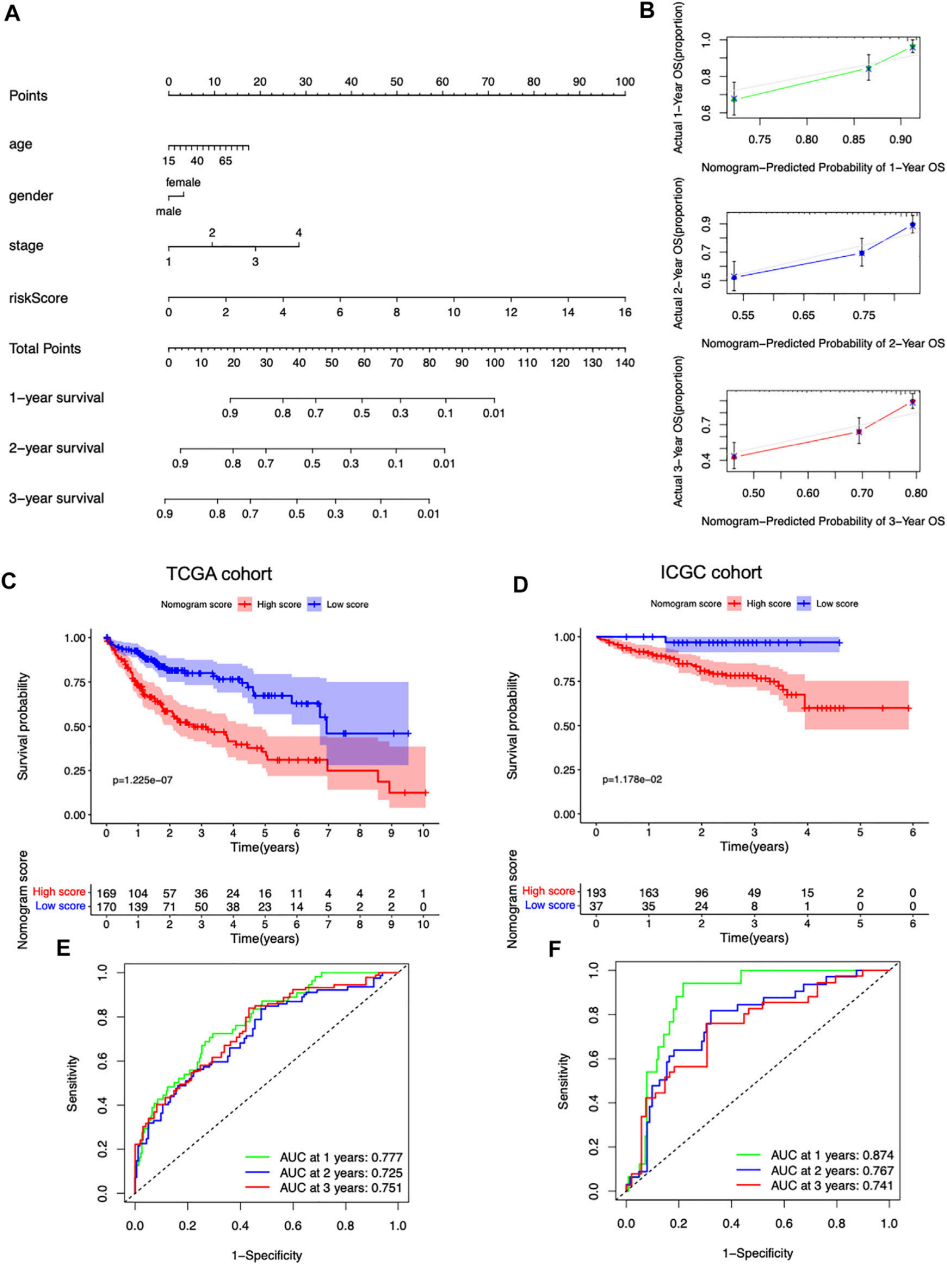
Predictive nomogram development and validation. **(A)** Nomogram based on the risk score and the clinical factors. **(B)** 1-, 2-, and 3-year calibration plots of the nomogram. **(C)** Survival curves stratified by the nomogram score in the TCGA cohort. **(D)** Survival curves stratified by the nomogram score in the ICGC cohort. **(E)** ROC analysis of the nomogram in the TCGA cohort. **(F)** ROC analysis of the nomogram in the ICGC cohort.

To facilitate the clinical applicability and availability of the seven-gene signature, a predictive nomogram for 1-, 2-, 3-year OS combined with age, gender, stage, and risk scores was developed (Figure 6A). The calibration curves showed that the nomogram had good prediction performance in HCC patients (Figure 6B). In the TCGA dataset, patients with a low nomogram score had significantly better survival than patients with a high nomogram score (*p* = 1.225e-07) (Figure 6C). The AUCs of the nomogram in the 1-, 2-, and 3-year ROC curves were 0.777, 0.725, and 0.751, which outperformed the seven-gene signature (Figure 6E). The nomogram was also validated in the ICGC dataset, and patients with a low score had a significantly better survival rate than those with a high score (*p* = 1.178e-02) (Figure 6D). The AUCs of the nomogram in the 1-, 2-, and 3-year ROC curves in the ICGC cohort were 0.874, 0.767, and 0.741, respectively (Figure 6F).

## Discussion

Nowadays, immune checkpoints have greatly changed the treatment paradigm and increased the survival of HCC patients (Nguyen et al., 2015). Traditional tumor markers including clinical tumor-node-metastasis (TNM) staging, vascular invasion, and other parameters help predict HCC prognosis (Bruix et al., 2016); however, these are gradually showing their limitations with the evolution of HCC management. Developing novel prediction models could guide patient prognostic stratification and facilitate personalized therapy.

In the present study, we systematically analyzed the expression of 568 cancer driver genes in the TCGA-LIHC cohort and found 189 differentially expressed cancer driver genes between cancer and normal tissues. We found that differentially expressed CDGs had high mutation rates in HCC, with TP53 showing the highest mutation rate (30%) followed by CTNNB1 (25%), TTN (24%), MUC16 (14%), ALB (13%), and PCLO (10%). These results indicated that the heterogeneity of HCC may be due to the diverse genetic abnormalities of cancer cells.

By univariate Cox regression analysis, 96 genes were found to be associated with the survival of HCC patients. LASSO regression and stepwise multivariate regression analyses found that a novel prognostic model comprising seven cancer driver genes was able to accurately distinguish HCC patients with different prognosis. Finally, a nomogram containing the clinical characteristics and genetic factors was constructed to provide a more accurate measure to predict the prognosis of HCC.

The prognostic signature that we constructed consisted of seven cancer driver genes (CDKN2C, HRAS, IRAK1, LOX, MYCN, NRAS, and PABPC1). These genes were all upregulated in the HCC tissues compared to normal tissues in the TCGA cohort. CDKN2C (cyclin-dependent kinase inhibitor 2C), also known as p18INK4C, is considered a tumor-suppressor gene (Gagrica et al., 2012). Dysregulated CDKN2C and its protease activity change are associated with the prognosis of HCC (Morishita et al., 2004). In human teratoma and thyroid tumor, mutant CDKN2C has been proven to predict poor prognosis (Cooke et al., 2017; El Naofal et al., 2017). Harvey-RAS (HRAS) and neuroblastoma-RAS (NRAS) belong to the RAS oncogene family. Sorafenib and regorafenib, the only effective therapeutic strategies for advanced HCC, target multiple kinase-related pathways including the RAS-RAF-ERK-pathway, underlining the crucial role of RAS signaling in HCC (Ostrem and Shokat, 2016; Pascual et al., 2016; Bruix et al., 2017). NRAS overexpression is demonstrated to be correlated with poor survival and sorafenib resistance in HCC (Dietrich et al., 2019). HRAS was also proven to be associated with the prognosis of HCC (Dietrich et al., 2018). IRAK1 is a widely expressed serine/threonine kinase, and phosphorylation of IRAK1 binds to the E3 ubiquitin ligase and TRAF6, leading to the activation of the NF-κB and MAPK pathways (Zhang and Ghosh, 2001; Flannery and Bowie, 2010). IRAK1 overexpression was proven to be correlated with metastasis and poor prognosis of HCC (Ye et al., 2017). LOX is a copper‐dependent amine oxidase that plays an important role in the formation of collagen and extracellular matrix (Erler et al., 2006). It is reported to be involved in the remodeling of cancer stroma and correlated to metastasis and dedifferentiation of cancer cells (Semenza, 2012; Boufraqech et al., 2016; Nilsson and Kannius-Janson, 2016). LOX overexpression can predict early recurrence and poor prognosis of HCC (Umezaki et al., 2019). MYCN, one of the members of the MYC family, plays crucial roles in regulating normal stem cell–mediated tissue regeneration and stem cell–mediated tumorigenesis (Dang, 2012; Qin et al., 2017). MYCN has been proven to be a prognostic biomarker and positively correlated with recurrence of *de novo* HCC after curative treatment (Qin et al., 2018). PABPC1 plays crucial roles in poly(A) shortening, recruitment of ribosome, and translation initiation *via* specifically binding to poly(A) tail of mRNA in cytoplasm (Kuhn and Wahle, 2004). High expression of PABPC1 has been proven to be correlated with worse overall survival for HCC (YuFeng and Ming, 2020). Taken together, these seven genes are closely related to the development and progression of cancer.

Immunotherapy using immune checkpoint inhibitors (ICIs) has dramatically changed the treatment of various malignancies. Anti-PD-1/L1 therapies, such as atezolizumab, pembrolizumab, and nivolumab, have shown promising benefits in a subset of HCC patients, alone or in combination with other agents (El-Khoueiry et al., 2017; Zhu et al., 2018; Finn et al., 2020a; Finn et al., 2020b). Screening out potential patients who may benefit from immunotherapy is the focus of research. In our study, the low-risk score group showed elevated expression of Type_1_IFN_Response and Type_2_IFN_Response signaling pathways compared to its counterpart. Moreover, it had more CD8+T cells and less M0 macrophages infiltrated. These results implied that the low-risk group might have a better immune microenvironment and responded better to immune checkpoint inhibitors. A nomogram consisting of the risk score and several clinical factors was constructed, which showed great accuracy to predict the prognosis of HCC patients.

There are some limitations in our study. First, the risk signature was built based on the TCGA-LIHC dataset and was only validated in the ICGC HCC dataset. Larger cohorts containing more patients are needed to verify the prognostic value of the risk score signature and nomogram. Second, the potential biological functions of genes contained in the risk signature have not been investigated. Further research should be conducted to elucidate the relevant mechanisms. Third, most HCC patients in the TCGA database were Caucasian, and it is not clear whether the risk signature has the same predictive effect in non-Caucasian races. However, our study provided an insight into the mutation landscape and expression pattern of the CDGs and constructed a risk score model and nomogram for prognosis prediction. This study highlighted the significance of CDGs in the HCC and provided a novel horizon for the investigation of HCC in the future.

## Data Availability

The original contributions presented in the study are included in the article/Supplementary Material; further inquiries can be directed to the corresponding author.
