# Supplementary material for: Comprehensive analysis of the cancer driver genes constructs a seven-gene signature for prediction of survival and tumor immunity in hepatocellular carcinoma
Source: Front Genet. 2022 Aug 9;13:937948. doi: 10.3389/fgene.2022.937948 (PMC9395598; doi:10.3389/fgene.2022.937948)
Supplement: Supplementary file 1 [file Table2.DOC]

#CDG matrix
rt=read.table("symbol.txt",sep="\t",header=T,check.names=F)
rt=as.matrix(rt)
rownames(rt)=rt[,1]
exp=rt[,2:ncol(rt)]
dimnames=list(rownames(exp),colnames(exp))
data=matrix(as.numeric(as.matrix(exp)),nrow=nrow(exp),dimnames=dimnames)
data=avereps(data)
data=data[rowMeans(data)>0,]
gene=read.table("gene.txt", header=F, check.names=F, sep="\t")
sameGene=intersect(as.vector(gene[,1]),rownames(data))
geneExp=data[sameGene,]
out=rbind(ID=colnames(geneExp),geneExp)
write.table(out,file="tcgaRBPexp.txt",sep="\t",quote=F,col.names=F)
#difference analysis
library(limma)
library(pheatmap)
fdrFilter=0.05                                        
logFCfilter=0.5                                        
conNum=                                           
treatNum=                                           
outTab=data.frame()
grade=c(rep(1,conNum),rep(2,treatNum))
rt=read.table(inputFile,sep="\t",header=T,check.names=F)
rt=as.matrix(rt)
rownames(rt)=rt[,1]
exp=rt[,2:ncol(rt)]
dimnames=list(rownames(exp),colnames(exp))
data=matrix(as.numeric(as.matrix(exp)),nrow=nrow(exp),dimnames=dimnames)
data=avereps(data)
data=data[rowMeans(data)>1,]
data=normalizeBetweenArrays(dataï¼‰
for(i in row.names(data)){
	geneName=unlist(strsplit(i,"\\|",))[1]
	geneName=gsub("\\/", "_", geneName)
	rt=rbind(expression=data[i,],grade=grade)
	rt=as.matrix(t(rt))
	wilcoxTest<-wilcox.test(expression ~ grade, data=rt)
	conGeneMeans=mean(data[i,1:conNum])
	treatGeneMeans=mean(data[i,(conNum+1):ncol(data)])
	logFC=log2(treatGeneMeans)-log2(conGeneMeans)
	pvalue=wilcoxTest$p.value
	conMed=median(data[i,1:conNum])
	treatMed=median(data[i,(conNum+1):ncol(data)])
	diffMed=treatMed-conMed
	if( ((logFC>0) & (diffMed>0)) | ((logFC<0) & (diffMed<0)) ){  
		outTab=rbind(outTab,cbind(gene=i,conMean=conGeneMeans,treatMean=treatGeneMeans,logFC=logFC,pValue=pvalue))
	}
}
pValue=outTab[,"pValue"]
fdr=p.adjust(as.numeric(as.vector(pValue)),method="fdr")
outTab=cbind(outTab,fdr=fdr)
outDiff=outTab[( abs(as.numeric(as.vector(outTab$logFC)))>logFCfilter & as.numeric(as.vector(outTab$fdr))<fdrFilter),]
write.table(outDiff,file="XX.xls",sep="\t",row.names=F,quote=F)
outDiff=outTab[( abs(as.numeric(as.vector(outTab$logFC)))>logFCfilter & as.numeric(as.vector(outTab$fdr))<fdrFilter),]
write.table(outDiff,file="XX.txt",sep="\t",row.names=F,quote=F)
up=outTab[(as.numeric(as.vector(outTab$logFC))>logFCfilter & as.numeric(as.vector(outTab$fdr))<fdrFilter),]
write.table(up,file="up.txt",sep="\t",row.names=F,quote=F)
down=outTab[(as.numeric(as.vector(outTab$logFC))< -logFCfilter & as.numeric(as.vector(outTab$fdr))<fdrFilter),]
write.table(down,file="down.txt",sep="\t",row.names=F,quote=F)
heatmap=rbind(ID=colnames(data[as.vector(outDiff[,1]),]),data[as.vector(outDiff[,1]),])
write.table(heatmap,file="tcgaDi2020p.txt",sep="\t",col.names=F,quote=F)
pdf(file="vol.pdf",height=5,width=5)
xMax=max(abs(as.numeric(as.vector(outTab$logFC))))
yMax=max(-log10(outTab$fdr))+1
plot(as.numeric(as.vector(outTab$logFC)), -log10(outTab$fdr), xlab="logFC",ylab="-log10(fdr)",
     main="Volcano", ylim=c(0,yMax),xlim=c(-xMax,xMax),yaxs="i",pch=20, cex=0.8)
diffSub=subset(outTab, fdr<fdrFilter & as.numeric(as.vector(logFC))>logFCfilter)
points(as.numeric(as.vector(diffSub$logFC)), -log10(diffSub$fdr), pch=20, col="red",cex=0.8)
diffSub=subset(outTab, fdr<fdrFilter & as.numeric(as.vector(logFC))<(-logFCfilter))
points(as.numeric(as.vector(diffSub$logFC)), -log10(diffSub$fdr), pch=20, col="green",cex=0.8)
abline(v=0,lty=2,lwd=3)
legend("topright", c("Down","Unchange","Up" ),bty="n",pch=19,col=c("green","black","red"),cex=0.8,title ="Significant")
dev.off()
hmExp=data[as.vector(outDiff[,1]),]
hmExp=log2(hmExp+0.1)
Type=c(rep("N",conNum),rep("T",treatNum))
names(Type)=colnames(data)
Type=as.data.frame(Type)
pdf(file="heatmap.pdf",height=7,width=10)
pheatmap(hmExp, 
         annotation=Type, 
         color = colorRampPalette(c("green", "black", "red"))(50),
         cluster_cols =F,
         show_colnames = F,
         show_rownames = F,
         scale="row",
         fontsize = 12,
         fontsize_row=3,
         fontsize_col=10)
dev.off()
#GO analysis
library("clusterProfiler")
library("org.Hs.eg.db")
library("enrichplot")
pvalueFilter=0.05        
qvalueFilter=0.05         
colorSel="qvalue"
if(qvalueFilter>0.05){
	colorSel="pvalue"
}
bioGO=function(input=null,goResult=null,GObar=null,GObub=null){
	rt=read.table(input,sep="\t",check.names=F,header=T)      
		genes=as.vector(rt[,1])
	entrezIDs=mget(genes, org.Hs.egSYMBOL2EG, ifnotfound=NA)
	entrezIDs=as.character(entrezIDs)
	gene=entrezIDs[entrezIDs!="NA"]                                
	kk <- enrichGO(gene = gene,
	               OrgDb = org.Hs.eg.db, 
	               pvalueCutoff=pvalueFilter, 
	               qvalueCutoff=qvalueFilter,
	               ont="all",
	               readable =T)
	write.table(kk,file=goResult,sep="\t",quote=F,row.names = F)                
	}

bioGO(input="diff.txt",goResult="GO.txt")
#KEGG analysis
library("clusterProfiler")
library("org.Hs.eg.db")
library("enrichplot")
pvalueFilter=0.05          
qvalueFilter=0.05        
colorSel="qvalue"
if(qvalueFilter>0.05){
	colorSel="pvalue"
}
bioKEGG=function(input=null,keggResult=null,KEGGbar=null,KEGGbub=null){
	rt=read.table(input,sep="\t",check.names=F,header=T)   
	genes=as.vector(rt[,1])
	entrezIDs=mget(genes, org.Hs.egSYMBOL2EG, ifnotfound=NA)
	entrezIDs=as.character(entrezIDs)
	rt=cbind(rt,entrezIDs)
	gene=entrezIDs[entrezIDs!="NA"]          
	kk <- enrichKEGG(gene = gene, organism = "hsa", pvalueCutoff =1, qvalueCutoff =1)  
	KEGG=as.data.frame(kk)
	KEGG$geneID=as.character(sapply(KEGG$geneID,function(x)paste(rt$gene[match(strsplit(x,"/")[[1]],as.character(rt$entrezID))],collapse="/")))
	KEGG=KEGG[(KEGG$pvalue<pvalueFilter & KEGG$qvalue<qvalueFilter),]
	write.table(KEGG,file=keggResult,sep="\t",quote=F,row.names = F)                         

	}
bioKEGG(input="diff.txt",keggResult="KEGG.txt")
#clinical and expression
library(limma)
expFile="tcgaRBPexp.share.txt"                               
cliFile="time.txt"                                             
rt=read.table(expFile,sep="\t",header=T,check.names=F)
rt=as.matrix(rt)
rownames(rt)=rt[,1]
exp=rt[,2:ncol(rt)]
dimnames=list(rownames(exp),colnames(exp))
data=matrix(as.numeric(as.matrix(exp)),nrow=nrow(exp),dimnames=dimnames)
data=avereps(data)
data=data[rowMeans(data)>0,]
group=sapply(strsplit(colnames(data),"\\-"),"[",4)
group=sapply(strsplit(group,""),"[",1)
group=gsub("2","1",group)
data=data[,group==0]
colnames(data)=gsub("(.*?)\\-(.*?)\\-(.*?)\\-(.*?)\\-.*","\\1\\-\\2\\-\\3",colnames(data))
data=t(data)
data=avereps(data)
cli=read.table(cliFile,sep="\t",check.names=F,header=T,row.names=1)    
sameSample=intersect(row.names(data),row.names(cli))
data=data[sameSample,]
cli=cli[sameSample,]
out=cbind(cli,data)
out=cbind(id=row.names(out),out)
write.table(out,file="TCGAExpTime.txt",sep="\t",row.names=F,quote=F)

# Cox and K-M 
library(survival)
coxPfilter=0.01                                           
kmPfilter=0.05                                           
rt=read.table("tcgaExpTime.txt",header=T,sep="\t",check.names=F,row.names=1)     
rt$futime=rt$futime/365
outTab=data.frame()
sigGenes=c("futime","fustat")
for(i in colnames(rt[,3:ncol(rt)])){
	cox <- coxph(Surv(futime, fustat) ~ rt[,i], data = rt)
	coxSummary = summary(cox)
	coxP=coxSummary$coefficients[,"Pr(>|z|)"]
	group=ifelse(rt[,i]>median(rt[,i]),"high","low")
	diff=survdiff(Surv(futime, fustat) ~group,data = rt)
	pValue=1-pchisq(diff$chisq,df=1)
	if((coxP<coxPfilter) & (pValue<kmPfilter)){
	    sigGenes=c(sigGenes,i)
			outTab=rbind(outTab,
			             cbind(id=i,
			             HR=coxSummary$conf.int[,"exp(coef)"],
			             HR.95L=coxSummary$conf.int[,"lower .95"],
			             HR.95H=coxSummary$conf.int[,"upper .95"],
			             pvalue=coxSummary$coefficients[,"Pr(>|z|)"])
			             )
	}
}
write.table(outTab,file="tcgaUniCox.txt",sep="\t",row.names=F,quote=F)
uniSigExp=rt[,sigGenes]
uniSigExp=cbind(id=row.names(uniSigExp),uniSigExp)
write.table(uniSigExp,file="tcgaUniSigExp.txt",sep="\t",row.names=F,quote=F)
rt <- read.table("tcgaUniCox.txt",header=T,sep="\t",row.names=1,check.names=F)
gene <- rownames(rt)
hr <- sprintf("%.3f",rt$"HR")
hrLow  <- sprintf("%.3f",rt$"HR.95L")
hrHigh <- sprintf("%.3f",rt$"HR.95H")
Hazard.ratio <- paste0(hr,"(",hrLow,"-",hrHigh,")")
pVal <- ifelse(rt$pvalue<0.001, "<0.001", sprintf("%.3f", rt$pvalue))
pdf(file="forest.pdf", width = 6,height = 4.5)
n <- nrow(rt)
nRow <- n+1
ylim <- c(1,nRow)
layout(matrix(c(1,2),nc=2),width=c(3,2))
xlim = c(0,3)
par(mar=c(4,2.5,2,1))
plot(1,xlim=xlim,ylim=ylim,type="n",axes=F,xlab="",ylab="")
text.cex=0.8
text(0,n:1,gene,adj=0,cex=text.cex)
text(1.5-0.5*0.2,n:1,pVal,adj=1,cex=text.cex);text(1.5-0.5*0.2,n+1,'pvalue',cex=text.cex,font=2,adj=1)
text(3,n:1,Hazard.ratio,adj=1,cex=text.cex);text(3,n+1,'Hazard ratio',cex=text.cex,font=2,adj=1,)
par(mar=c(4,1,2,1),mgp=c(2,0.5,0))
xlim = c(0,max(as.numeric(hrLow),as.numeric(hrHigh)))
plot(1,xlim=xlim,ylim=ylim,type="n",axes=F,ylab="",xaxs="i",xlab="Hazard ratio")
arrows(as.numeric(hrLow),n:1,as.numeric(hrHigh),n:1,angle=90,code=3,length=0.05,col="darkblue",lwd=2.5)
abline(v=1,col="black",lty=2,lwd=2)
boxcolor = ifelse(as.numeric(hr) > 1, 'red', 'green')
points(as.numeric(hr), n:1, pch = 15, col = boxcolor, cex=1.3)
axis(1)
dev.off()
#lasson
library("glmnet")
library("survival")
rt=read.table("tcgauniSigExp.txt",header=T,sep="\t",row.names=1,check.names=F)     
x=as.matrix(rt[,c(3:ncol(rt))])
y=data.matrix(Surv(rt$futime,rt$fustat))
fit <- glmnet(x, y, family = "cox", maxit = 1000)
pdf("lambda.pdf")
plot(fit, xvar = "lambda", label = TRUE)
dev.off()
cvfit <- cv.glmnet(x, y, family="cox", maxit = 1000)
pdf("cvfit.pdf")
plot(cvfit)
abline(v=log(c(cvfit$lambda.min,cvfit$lambda.1se)),lty="dashed")
dev.off()
coef <- coef(fit, s = cvfit$lambda.min)
index <- which(coef != 0)
actCoef <- coef[index]
lassoGene=row.names(coef)[index]
lassoGene=c("futime","fustat",lassoGene)
lassoSigExp=rt[,lassoGene]
lassoSigExp=cbind(id=row.names(lassoSigExp),lassoSigExp)
write.table(lassoSigExp,file="lassoSigExp.txt",sep="\t",row.names=F,quote=F)

#stepwise Cox 
library(glmnet)
library(survival)         
rt=read.table("tcgaUniSigExp.txt",header=T,sep="\t",row.names=1)   
multiCox=coxph(Surv(futime, fustat) ~ ., data = rt)
multiCox=step(multiCox,direction = "both")
multiCoxSum=summary(multiCox)
outMultiTab=data.frame()
outMultiTab=cbind(
		          coef=multiCoxSum$coefficients[,"coef"],
		          HR=multiCoxSum$conf.int[,"exp(coef)"],
		          HR.95L=multiCoxSum$conf.int[,"lower .95"],
		          HR.95H=multiCoxSum$conf.int[,"upper .95"],
		          pvalue=multiCoxSum$coefficients[,"Pr(>|z|)"])
outMultiTab=cbind(id=row.names(outMultiTab),outMultiTab)
write.table(outMultiTab,file="multiCox.txt",sep="\t",row.names=F,quote=F)
trainScore=predict(multiCox,type="risk",newdata=rt)
coxGene=rownames(multiCoxSum$coefficients)
coxGene=gsub("`","",coxGene)
outCol=c("futime","fustat",coxGene)
risk=as.vector(ifelse(trainScore>median(trainScore),"high","low"))
outTab=cbind(rt[,outCol],riskScore=as.vector(trainScore),risk)
write.table(cbind(id=rownames(outTab),outTab),file="trainRisk.txt",sep="\t",quote=F,row.names=F)
rt=read.table("ICGCExpTime.txt",header=T,sep="\t",row.names=1)
testFinalGeneExp=rt[,coxGene]
testScore=predict(multiCox,type="risk",newdata=rt)
outCol=c("futime","fustat",coxGene)
risk=as.vector(ifelse(testScore>median(trainScore),"high","low"))
outTab=cbind(rt[,outCol],riskScore=as.vector(testScore),risk)
write.table(cbind(id=rownames(outTab),outTab),file="testRisk.txt",sep="\t",quote=F,row.names=F)

#Survival
library(survival)
library(survminer)
bioSurvival=function(inputFile=null,outFile=null){
	rt=read.table(inputFile,header=T,sep="\t")
	diff=survdiff(Surv(futime, fustat) ~risk,data = rt)
	pValue=1-pchisq(diff$chisq,df=1)
	pValue=signif(pValue,4)
	pValue=format(pValue, scientific = TRUE)
	fit <- survfit(Surv(futime, fustat) ~ risk, data = rt)
	surPlot=ggsurvplot(fit, 
		           data=rt,
		           conf.int=T,
		           pval=paste0("p=",pValue),
		           pval.size=4,
		           risk.table=TRUE,
		           legend.labs=c("High risk", "Low risk"),
		           legend.title="Risk",
		           xlab="Time(years)",
		           break.time.by = 2,
		           risk.table.title="",
		           palette=c("red", "blue"),
		           risk.table.height=0.2)
	pdf(file=outFile,onefile = FALSE,width = 6.5,height =5.5)
	print(surPlot)
	dev.off()
}
bioSurvival(inputFile="Risk.txt",outFile="Surv.pdf")

#library(pheatmap)
bioRiskPlot=function(inputFile=null,riskScoreFile=null,survStatFile=null,heatmapFile=null){
	rt=read.table(inputFile,sep="\t",header=T,row.names=1,check.names=F)   
	rt=rt[order(rt$riskScore),]                                            

	riskClass=rt[,"risk"]
	lowLength=length(riskClass[riskClass=="low"])
	highLength=length(riskClass[riskClass=="high"])
	line=rt[,"riskScore"]
	line[line>10]=10
	pdf(file=riskScoreFile,width = 10,height = 3.5)
	plot(line, type="p", pch=20,
		 xlab="Patients (increasing risk socre)", ylab="Risk score",
		 col=c(rep("green",lowLength),rep("red",highLength)) )
	abline(h=median(rt$riskScore),v=lowLength,lty=2)
	legend("topleft", c("High risk", "low Risk"),bty="n",pch=19,col=c("red","green"),cex=1.2)
	dev.off()
	color=as.vector(rt$fustat)
	color[color==1]="red"
	color[color==0]="green"
	pdf(file=survStatFile,width = 10,height = 3.5)
	plot(rt$futime, pch=19,
		 xlab="Patients (increasing risk socre)", ylab="Survival time (years)",
		 col=color)
	legend("topleft", c("Dead", "Alive"),bty="n",pch=19,col=c("red","green"),cex=1.2)
	abline(v=lowLength,lty=2)
	dev.off()
	rt1=rt[c(3:(ncol(rt)-2))]
	rt1=log2(rt1+1)
	rt1=t(rt1)
	annotation=data.frame(type=rt[,ncol(rt)])
	rownames(annotation)=rownames(rt)
	pdf(file=heatmapFile,width = 10,height = 3.5)
	pheatmap(rt1, 
		     annotation=annotation, 
		     cluster_cols = FALSE,
		     fontsize_row=11,
		     show_colnames = F,
		     fontsize_col=3,
		     color = colorRampPalette(c("green", "black", "red"))(50) )
	dev.off()
}
bioRiskPlot(inputFile=Risk.txt",riskScoreFile="riskScore.pdf",survStatFile="survStat.pdf",heatmapFile="heatmap.pdf")

#riskscore independent analysis
risk=read.table("Riskclinical.txt",header=T,sep="\t",check.names=F,row.names=1) 
uniTab=data.frame()
for(i in colnames(rt[,3:ncol(rt)])){
	 cox <- coxph(Surv(futime, fustat) ~ rt[,i], data = rt)
	 coxSummary = summary(cox)
	 uniTab=rbind(uniTab,
	              cbind(id=i,
	              HR=coxSummary$conf.int[,"exp(coef)"],
	              HR.95L=coxSummary$conf.int[,"lower .95"],
	              HR.95H=coxSummary$conf.int[,"upper .95"],
	              pvalue=coxSummary$coefficients[,"Pr(>|z|)"])
	              )
}
write.table(uniTab,file="train.uniCox.txt",sep="\t",row.names=F,quote=F)
multiCox=coxph(Surv(futime, fustat) ~ ., data = rt)
multiCoxSum=summary(multiCox)
multiTab=data.frame()
multiTab=cbind(
             HR=multiCoxSum$conf.int[,"exp(coef)"],
             HR.95L=multiCoxSum$conf.int[,"lower .95"],
             HR.95H=multiCoxSum$conf.int[,"upper .95"],
             pvalue=multiCoxSum$coefficients[,"Pr(>|z|)"])
multiTab=cbind(id=row.names(multiTab),multiTab)
write.table(multiTab,file="train.multiCox.txt",sep="\t",row.names=F,quote=F)

#nomogram
library(rms)
riskFile="Risk.txt"    
outFile="Nomogram.pdf"     
risk=read.table(riskFile,header=T,sep="\t",check.names=F,row.names=1)      
rt=risk[,1:(ncol(risk)-2)]
dd <- datadist(rt)
options(datadist="dd")
f <- cph(Surv(futime, fustat) ~ ., x=T, y=T, surv=T, data=rt, time.inc=1)
surv <- Survival(f)
nom <- nomogram(f, fun=list(function(x) surv(1, x), function(x) surv(2, x), function(x) surv(3, x)), 
    lp=F, funlabel=c("1-year survival", "2-year survival", "3-year survival"), 
    maxscale=100, 
    fun.at=c(0.99, 0.9, 0.8, 0.7, 0.5, 0.3,0.1,0.01))  
pdf(file=outFile,height=6,width=9)
plot(nom)
dev.off()
#calibration 
time=3   
f <- cph(Surv(futime, fustat) ~ ., x=T, y=T, surv=T, data=rt, time.inc=time)
cal <- calibrate(f, cmethod="KM", method="boot", u=time, m=100, B=1000)
pdf(file="calibration.pdf",height=6,width=8)
plot(cal,xlab="Nomogram of 3-Year",ylab="3-Year",col="red",sub=F)
dev.off()
